# Antidepressant-like effects and cognitive enhancement of Schisandra chinensis in chronic unpredictable mild stress mice and its related mechanism

**DOI:** 10.1038/s41598-017-07407-1

**Published:** 2017-07-31

**Authors:** Tingxu Yan, Bosai He, Shutong Wan, Mengjie Xu, Huilin Yang, Feng Xiao, Kaishun Bi, Ying Jia

**Affiliations:** 1 School of Traditional Chinese Materia Medica, Shenyang Pharmaceutical University, Wenhua Road 103, Shenyang, 110016 China; 2 School of Pharmacy, Shenyang Pharmaceutical University, Wenhua Road 103, Shenyang, 110016 China; 3 School of Functional Food and Wine, Shenyang Pharmaceutical University, Wenhua Road 103, Shenyang, 110016 China

## Abstract

The aim of this study was to evaluate whether Schisandra chinensis extract (SCE) administration influences chronic unpredictable mild stress (CUMS)-induced depression and cognitive impairment, and explores underlying mechanisms. Sucrose preference test (SPT) and forced swimming test (FST) were used for assessing depressive symptoms, and Y-maze, Morris water maze were used for evaluating cognition processes. The results showed that CUMS (4 weeks) was effective in producing both depression and memory deficits in mice. Additionally, CUMS exposure significantly decreased brain derived neurotrophic factor (BDNF) levels in hippocampus as indicated by ELISA, immunohistochemistry and immunofluorescence assays, accompanied by down-regulated tyrosine kinase receptor B (TrkB)/cAMP-response element binding protein (CREB)/extracellular signal-regulated kinase (ERK) and phosphatidylinositol 3 kinase (PI3K)/ protein kinase B (AKT)/ glycogen synthase kinase-3β (GSK-3β) signaling pathways. Chronic administration of SCE (600 or 1200 mg/kg, i.g.) significantly prevented all these CUMS-induced behavioral and biochemical alterations. It suggested that SCE could improve the depression-like emotional status and associated cognitive deficits in CUMS mice, which might be mediated by regulation of BDNF levels in hippocampus, as well as up-regulating of TrkB/CREB/ERK and PI3K/AKT/GSK-3β pathways.

## Introduction

Depression is a common illness worldwide, with an estimated 350 million people of all ages affected according to a report of WHO in 2014^1^, it is also the leading cause of disability and is a major contributor to the overall global burden of disease. Depression is a heterogeneous syndrome and may result in poorer well-being. At its worst, depression can lead to suicide^2^. In general, most clinical symptoms of major depressive disorder, such as delusions, anxiety, irritability or insomnia, can be effectively treated by current psychopharmacological treatments. Nevertheless, cognitive deficits (like a diminished ability to think or concentrate, or indecisiveness), which represent core deficits of depression^3^, may persist in patients even when depressive symptoms have abated or disappeared and significantly affect the individual’s social and occupational function. Indeed, meta-analyses have shown that cognitive deficits are still present in remitted patients^4^. For this reason, cognitive impairment emerges as a potential target for both pharmacological and psychosocial treatments, with the final goal of improving functioning.

BDNF is a member of the nerve growth factor family and is expressed in the adult mammalian central and peripheral nervous system, particularly in the hippocampus and cortex^5^. The vast majority of the literature linking neurotrophins to mood disorders deals with BDNF, other neurotrophins showing only very minor role^6–8^. In addition, clinical studies have shown that serum BDNF levels and hippocampal volume reductions in elderly individuals are closely correlated with memory loss^9, 10^, and BDNF may rescue cognitive impairments and learning deficits in Alzheimer’s disease^11, 12^. Previous reports indicated that changes in BDNF level are implicated in the pathophysiology of cognitive decline in depression and neurodegenerative disorders^13^. BDNF exerts its pro-survival effects by binding its high affinity receptor TrkB, to activate BDNF-TrkB signaling^14^. Behavioral responses to antidepressants have been abolished in animals in which BDNF-TrkB signaling is inhibited^15^. It is also involved in propofol-induced learning and memory impairments^16^. Thus, these results suggest that BDNF-TrkB signaling plays a critical role in the molecular mechanisms of antidepressants and cognition enhancement. BDNF-TrkB downstream signaling, including ERK/CREB and PI3K/AKT/GSK-3β pathways, can modulate neurotransmitter release, cell viability, apoptosis and postsynaptic responses, and these functions are closely associated with depression^17, 18^ and learning ability^19^.

About 30% of patients suffering from a major depressive disorder do not respond sufficiently to establish pharmacological, psychotherapeutic, or somatic treatments^20^. In addition, different studies conclude that only 30–40% of patients that are optimally treated with first line antidepressants achieve remission and more than one third of patients with depression are classified as treatment-resistant depression (TRD)^21^. In order to deal with the mentioned limitations shown by current antidepressant drugs, traditional Chinese medicine (TCM) is attracting increasing attention as a method for meeting the demands for higher remission rate, faster onset, persistent antidepressant action, and fewer adverse effects^22–24^. *Schisandra chinensis* (Trucz.) Bail. is a famous traditional Chinese medicine which is usually used in the clinic with the functions of inducing astringency, replenishing and promoting the production of body fluid and tonifying the kidneys to relieve mental strain^25^. Dibenzo[a,c]cyclooctadiene lignans are the main chemical components in Schisandra chinensis extract (SCE), and these are showing various pharmacological activities, of which their antioxidative, neuroprotective, anti-cancer, vasorelaxant and cytoprotective properties are among the most studied ones^26–28^. Our group previous reported that SCE could produce antidepressant-like effects in different depressed models^29, 30^, and ameliorate cognition deficits^31^. However, the potential neuroprotective effects of SCE against the CUMS-induced depression and cognitive deficits, as well as the mechanisms remain to be clarified. Thus, in this study, we used the CUMS model to investigate the antidepressant-like activity and cognitive impairment effects of SCE, moreover, the BDNF/TrkB/ERK/CREB and PI3K/AKT/GSK-3β signaling pathways were determined to illustrate the action mechanism of SCE.

## Results

### Effects of SCE on the sucrose preference in the SPT

**Figure 1:**
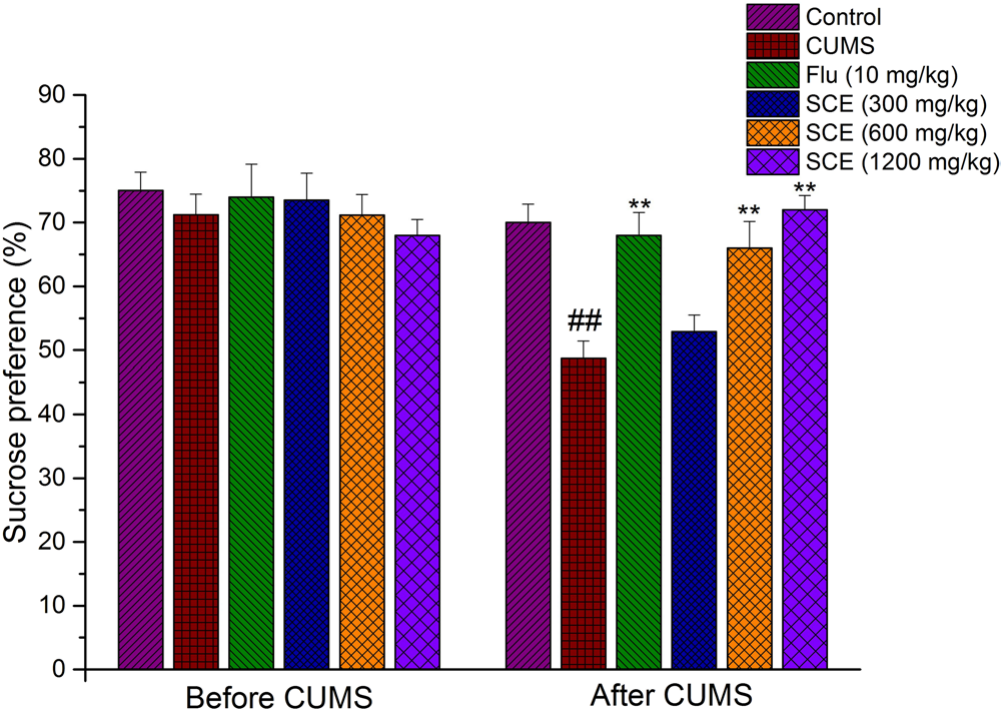
Effects of SCE administration on the sucrose preference in the SPT. The data represented the values of mean ± S.E.M. from 10 mice/group. **p < 0.01 vs. CUMS group. ^##^p < 0.01 vs. Control group.

As shown in Fig. 1, *Post hoc* analysis revealed that a 4-weeks CUMS exposure significantly reduced the percentage of sucrose consumption in the stressed mice in comparison with the control group (p < 0.01). However, long-term treatment with SCE (600 mg/kg or 1200 mg/kg) increased the sucrose preference as compared to the CUMS control mice (p < 0.01, p < 0.01, respectively). As one approach to investigate the antidepressant effects of SCE, the sucrose preference test, an indicator of anhedonia, was applied after 4 weeks of CUMS exposure. Such increases in sucrose consumption suggest a potent antidepressant-like effect of SCE within these CUMS-exposed mice.

### Effects of SCE on locomotor activity

There is no significance among all groups in the total travel distance which indicated that CUMS and drug treatment did not alter the locomotor activity of the test animal (Table 1).

**Table 1:** Influence of administration of SCE and CUMS procedure on the locomotor activity in mice.

Groups	locomotion length (cm/5 min)
Control	1236 ± 29
CUMS	1017 ± 57
Fluoxetine (10 mg/kg)	1138 ± 76
SCE (300 mg/kg)	995 ± 79
SCE (600 mg/kg)	1205 ± 84
SCE (1200 mg/kg)	1099 ± 59

### Effects of SCE on the immobility time in the FST

**Figure 2:**
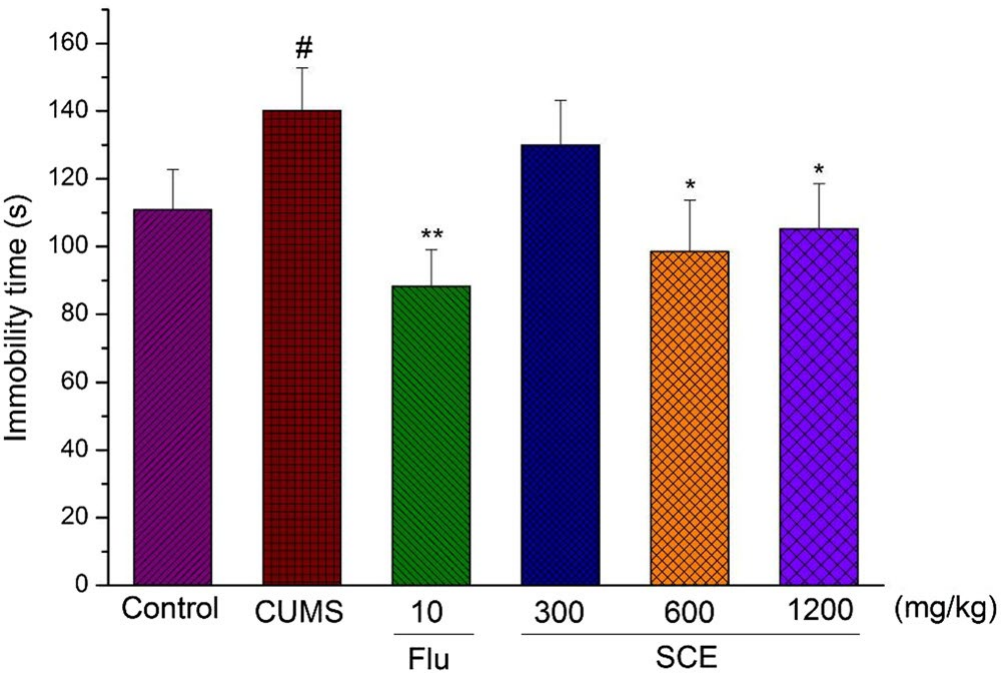
Effects of SCE administration on the imobility time in the FST. The data represented the values of mean ± S.E.M. from 10 mice/group. *p < 0.05, **p < 0.01 vs. Control group. ^#^p < 0.05 vs. Control group.

Consistent with the sucrose consumption test, *Post hoc* analysis indicated that 4-weeks of CUMS exposure significantly increased immobility times compared to the control mice (Fig. 2). These effects were reversed by chronic SCE treatment (600 mg/kg or 1200 mg/kg), with immobility durations now being significantly decreased as compared to the CUMS control group with regard to immobility time (p < 0.01, p < 0.01, respectively). These results complement those of the sucrose preference test, again showing an antidepressant-like effect of SCE as based upon these decreased immobility times in this FST.

### Effects of SCE on Y maze test

**Figure 3:**
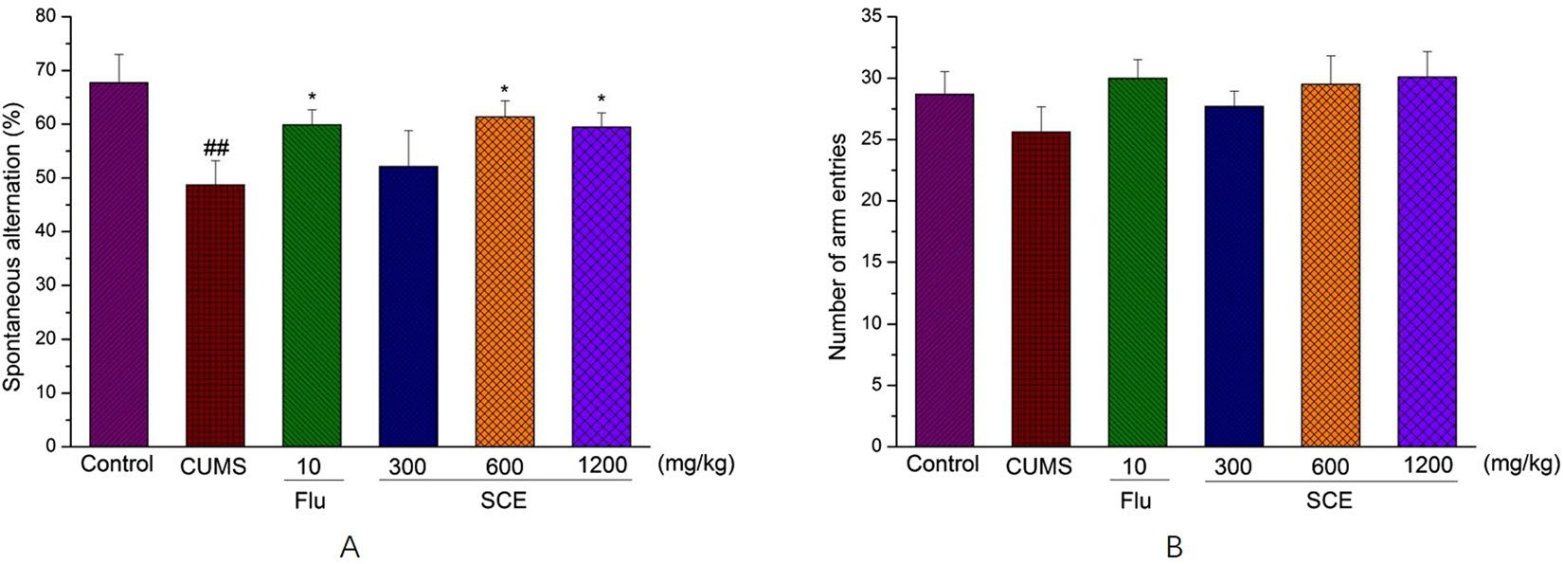
Effects of SCE administration on Y-maze test, spontaneous alternations (**A**), number of arm entries (**B**). The data represented the values of mean ± S.E.M. from 10 mice/group. *p < 0.05 vs. CUMS group. ^##^p < 0.01 vs. Control group.

Analyses of the spontaneous alternation percentage with in Y maze task showed significant overall differences between all groups (Fig. 3). Both doses of SCE (600 mg/kg or 1200 mg/kg), but especially 600 mg/kg, significantly improved memory formation in CUMS-induced mice as compared to the control mice. The changes in the spontaneous alternation percentages of CUMS-induced mice exposed to SCE are not related to the changes in motor activity, as evidenced by the number of arm entries as compared to the control mice.

### Effects of SCE on Morris water maze

A significant decrease of the escape latency to reach the submerged platform was evidenced along the 5 days of a training period in all the groups in the MWM test (Table 2). The CUMS group took more time on the last two training days to reach the platform compared with the control group in the place navigation test (p < 0.05). However, the SCE (600 or 1200 mg/kg) treated group significantly ameliorated the effects of CUMS on escape latency (p < 0.05, p < 0.05, p < 0.05 for the 3^rd^, 4^th^ and 5^th^ day, respectively). In the probe trial, a probe test was conducted by removing the platform on the last day of MWM task. The effects of SCE treatment on the performance of spatial probe trial in mice are shown in Fig. 4. Mice in the control group focus more time in the target quadrant, while mice in the CUMS group failed to remember the precise location of the platform. Treatment with SCE (600 or 1200 mg/kg) reversed the cognitive deficit as compared to the CUMS group.

**Table 2:** Effect of SCE on the escape latency (s) of CUMS-induced mice in water maze test.

Groups	Day 1	Day 2	Day 3	Day 4	Day 5
Control	83.91 ± 8.05	74.52 ± 9.87	63.00 ± 9.39	42.57 ± 5.71	23.62 ± 6.92
CUMS	85.49 ± 7.20	81.44 ± 9.76	72.94 ± 8.47	57.91 ± 9.55^#^	37.49 ± 8.25^#^
Flu (10)	88.94 ± 10.55	69.98 ± 6.63	62.04 ± 13.77	46.24 ± 11.05*	34.13 ± 7.39
SCE (300)	80.59 ± 6.36	77.14 ± 10.21	58.74 ± 7.76	49.07 ± 7.28	35.14 ± 8.99
SCE (600)	85.64 ± 9.01	71.47 ± 7.68	56.44 ± 10.49*	33.53 ± 10.19*	19.75 ± 6.18*
SCE (1200)	80.39 ± 10.65	75.40 ± 9.69	57.74 ± 4.34*	21.49 ± 9.45**	21.49 ± 11.03*

#p < 0.05, compared with control group; *p < 0.05, **p < 0.01, compared with CUMS group.

**Figure 4:**
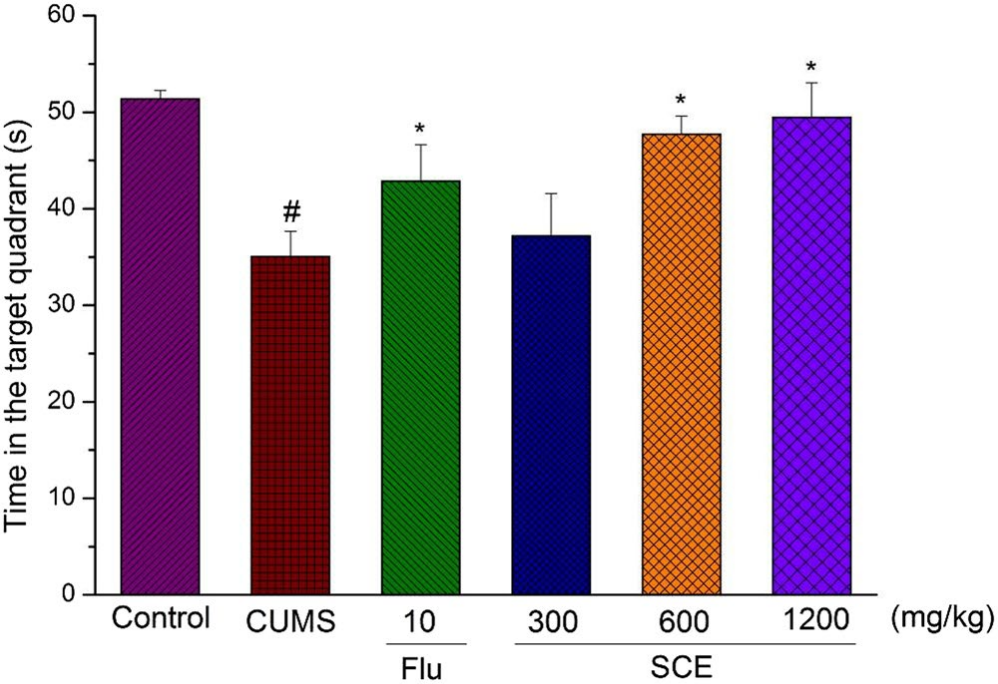
Effects of SCE administration on time in the target quadrant of Morris water maze. The data represented the values of mean ± S.E.M. from 10 mice/group. *p < 0.05 vs. CUMS group. ^##^p < 0.01 vs. Control group.

### Effects of SCE on BDNF/TrkB/CREB/ERK signaling pathways

**Figure 5:**
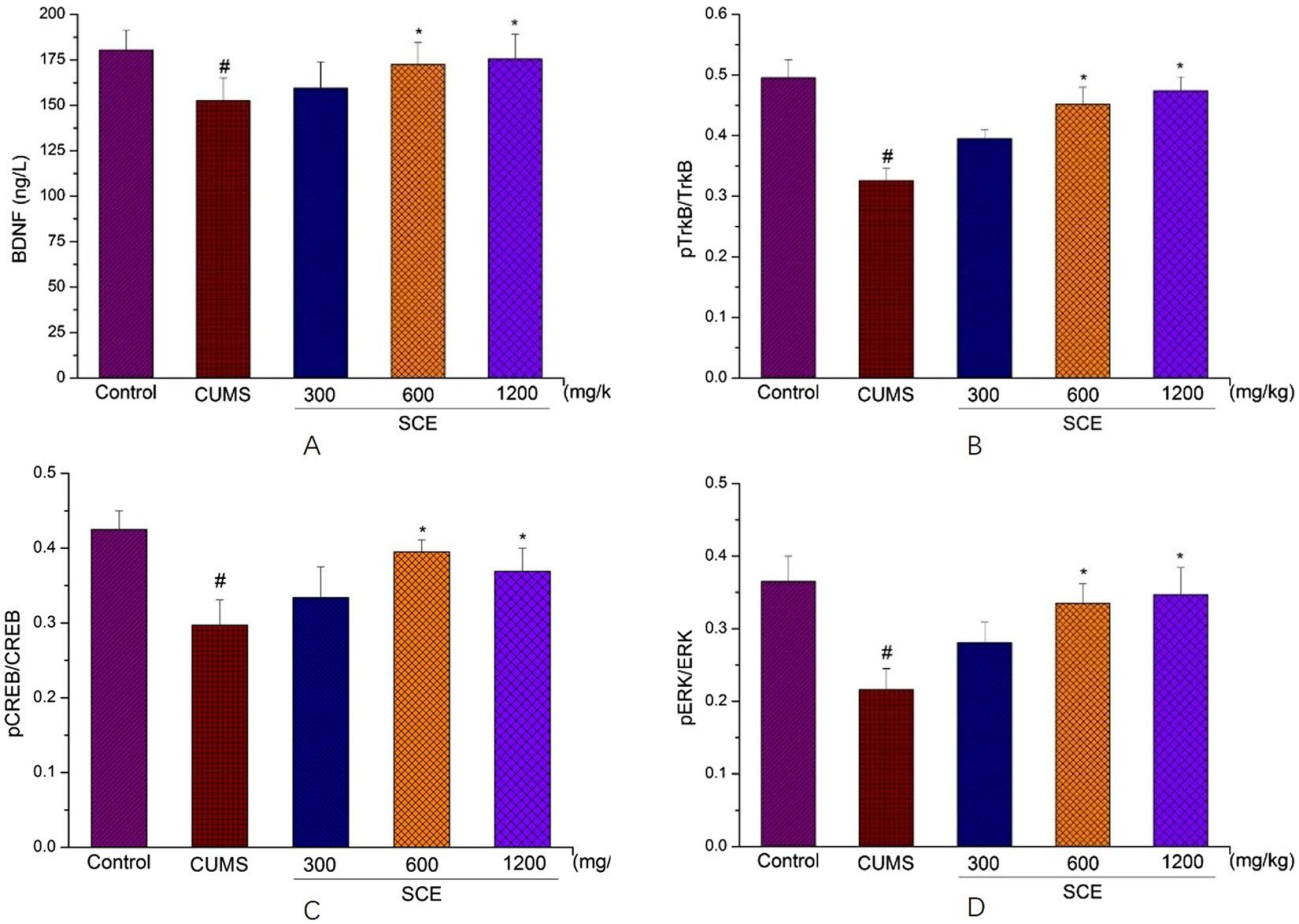
Effects of SCE administration on BDNF (**A**), pTrkB/TrkB (**B**), pCREB/CREB (**C**) and pERK/ERK (**D**) levels in hippocampus. The data represented the values of mean ± S.E.M. from 10 mice/group. *p < 0.05 vs. CUMS group. ^#^p < 0.05 vs. Control group.

Compared with the control group, the CUMS procedure could significantly decrease the levels of BDNF, pTrkB/TrkB, pCREB/CREB and pERK/ERK in hippocampus (p < 0.05). Meanwhile, SCE 600 mg/kg and 1200 mg/kg could dramatically upregulate the BDNF/TrkB/CREB/ERK signaling in the hippocampus of drug treated mice compared to the CUMS mice (p < 0.05) (Fig. 5A–D).

### Effects of SCE on BDNF in the mouse hippocampus by immunohistochemistry analysis

**Figure 6:**
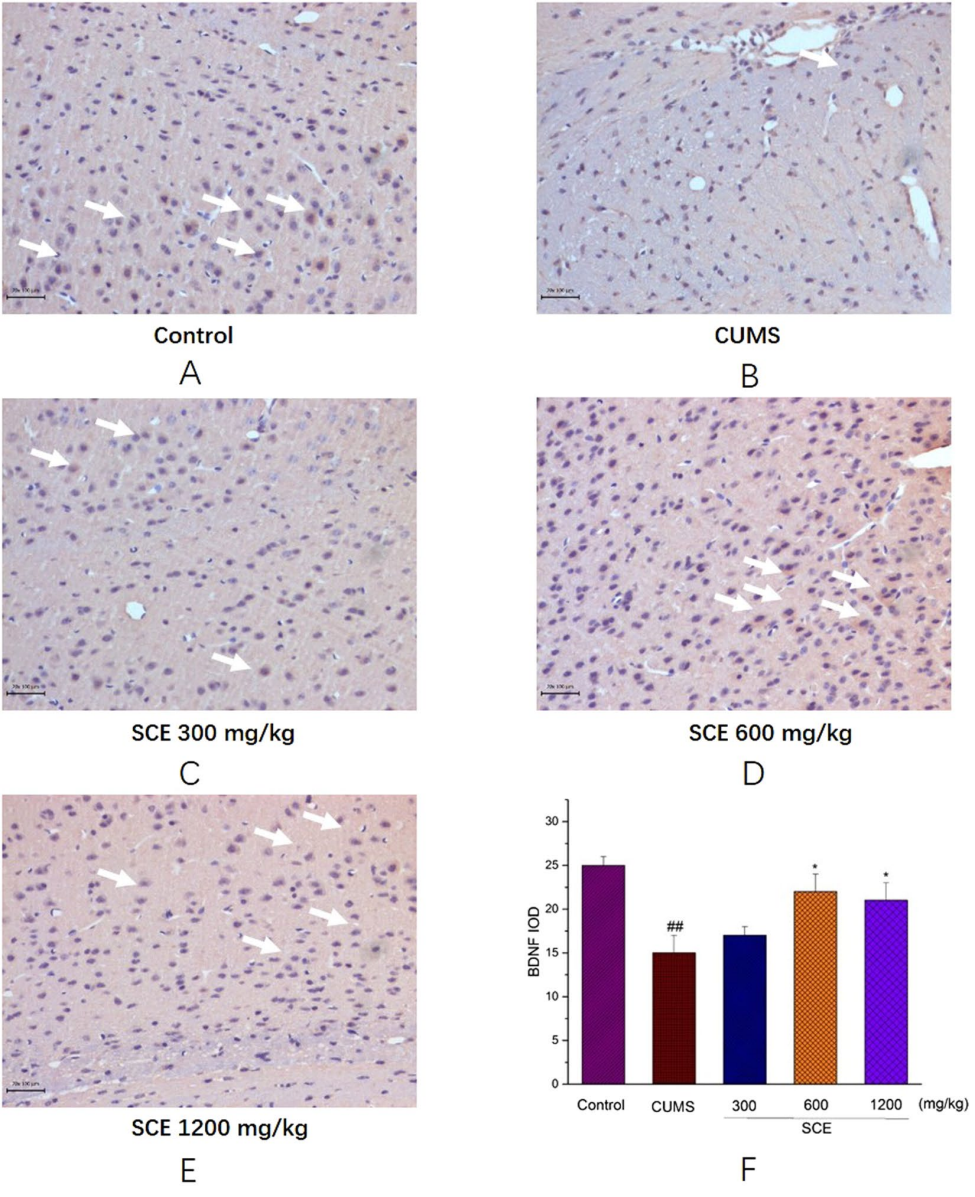
Immunohistochemistry of BDNF protein in the mice hippocampus, Control (**A**), CUMS (**B**), SCE 300 mg/kg (**C**), 600 mg/kg (**D**), 1200 mg/kg (**E**) and IOD of BDNF (**F**). The scale bar shows 100 μm. The data represented the values of mean ± S.E.M. from 10 mice/group. *p < 0.05 vs. CUMS group. ^##^p < 0.01 vs. Control group.

As shown in Fig. 6A–F, intergral optical density (IOD) of BDNF (brown particles) in CUMS group was remarkably lower than that in control group (p < 0.01). The number of positive hippocampal neurons and IOD in SCE (600 or 1200 mg/kg) were significantly higher than those in CUMS group (p < 0.05), indicating that SCE up-regulated the BDNF expression in hippocampal neurons which was consistent with the data of ELISA.

### Effects of SCE on BDNF in the mouse hippocampus

**Figure 7:**
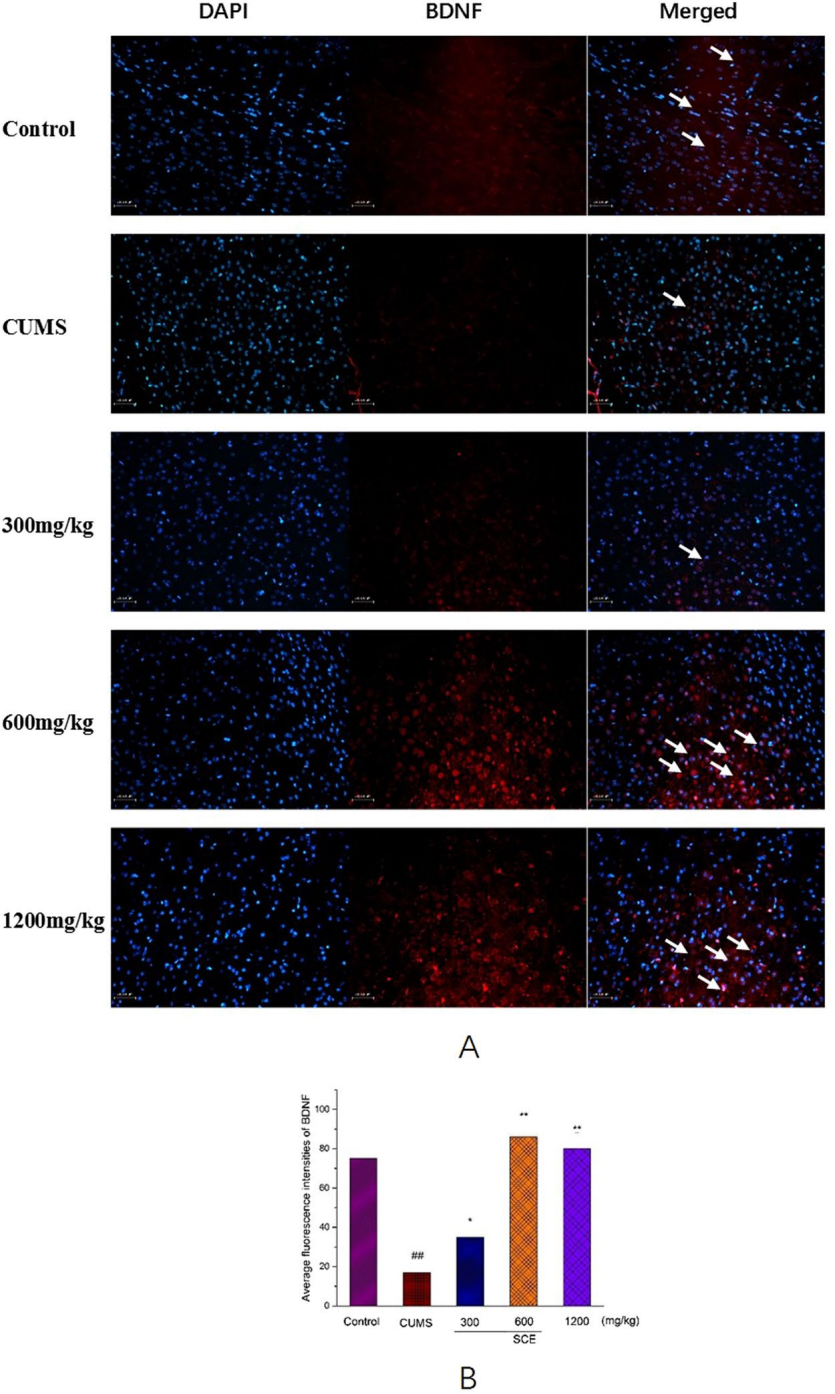
Effects of SCE on the expression of the immunoreactivity of BDNF in the mouse hippocampus. (**A**) Hippocampus were stained with specific antibodies against BDNF (red). Nuclei were stained with DAPI (blue). The scale bar shows 100 μm. (**B**) The average fluorescence intensities of BDNF were quantified after SCE treatment. The data represented the values of mean ± S.E.M. from 10 mice/group. *p < 0.05, **p < 0.01 vs. CUMS group. ^##^p < 0.01 vs. Control group.

Immunofluorescence assay showed that BDNF (red fluorescence, Fig. 7A) in depression mice was significantly lower compared with control mice. And SCE (600 or 1200 mg/kg) group could apparently increase the level of BDNF protein in comparison of the CUMS group were shown in Fig. 7B based on the average fluorescence intensities of BDNF. The results were in line with the results of ELISA and immunohistochemistry assay.

### Effects of SCE on PI3K/AKT/GSK-3β signaling pathways

**Figure 8:**
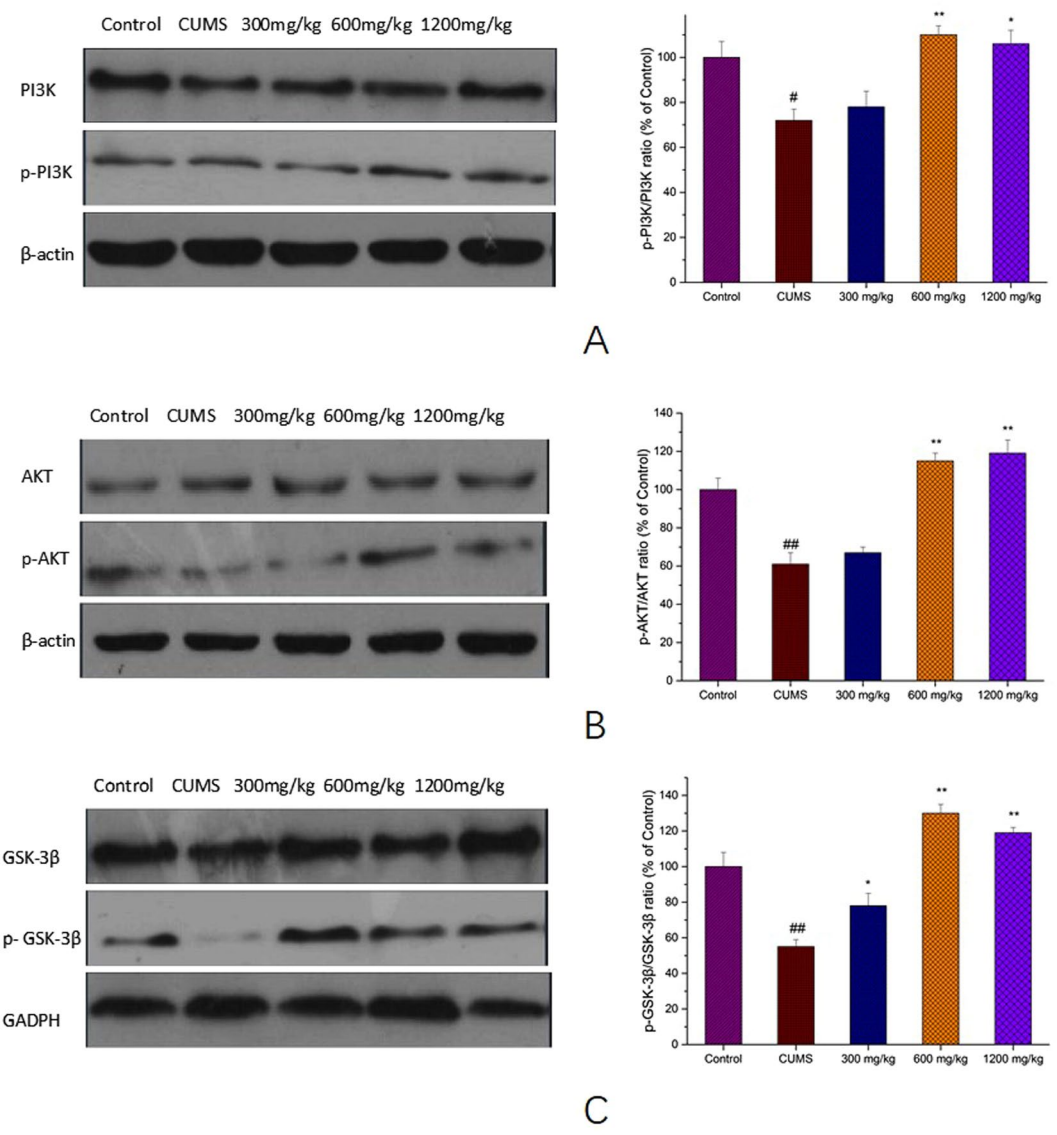
Effects of SCE administration on pPI3K/PI3K (**A**), pAKT/AKT (**B**) and p GSK-3β/ GSK-3β (**C**) levels in hippocampus. The data represented the values of mean ± S.E.M. from 10 mice/group. *p < 0.05, **p < 0.01 vs. CUMS group. ^#^p < 0.05, ^##^p < 0.01 vs. Control group.

Western blotting results (Fig. 8) indicated that PI3K was reduced in depression mice (p < 0.05) and this tendency was reversed by SCE (600 or 1200 mg/kg) significantly (p < 0.05). The data of AKT and GSK-3β turned out the same, which indicated that PI3K/AKT/GSK-3β might be involved in the antidepressant-like effect of SCE.

### Effects of SCE on histopathological changes in hippocampus

**Figure 9:**
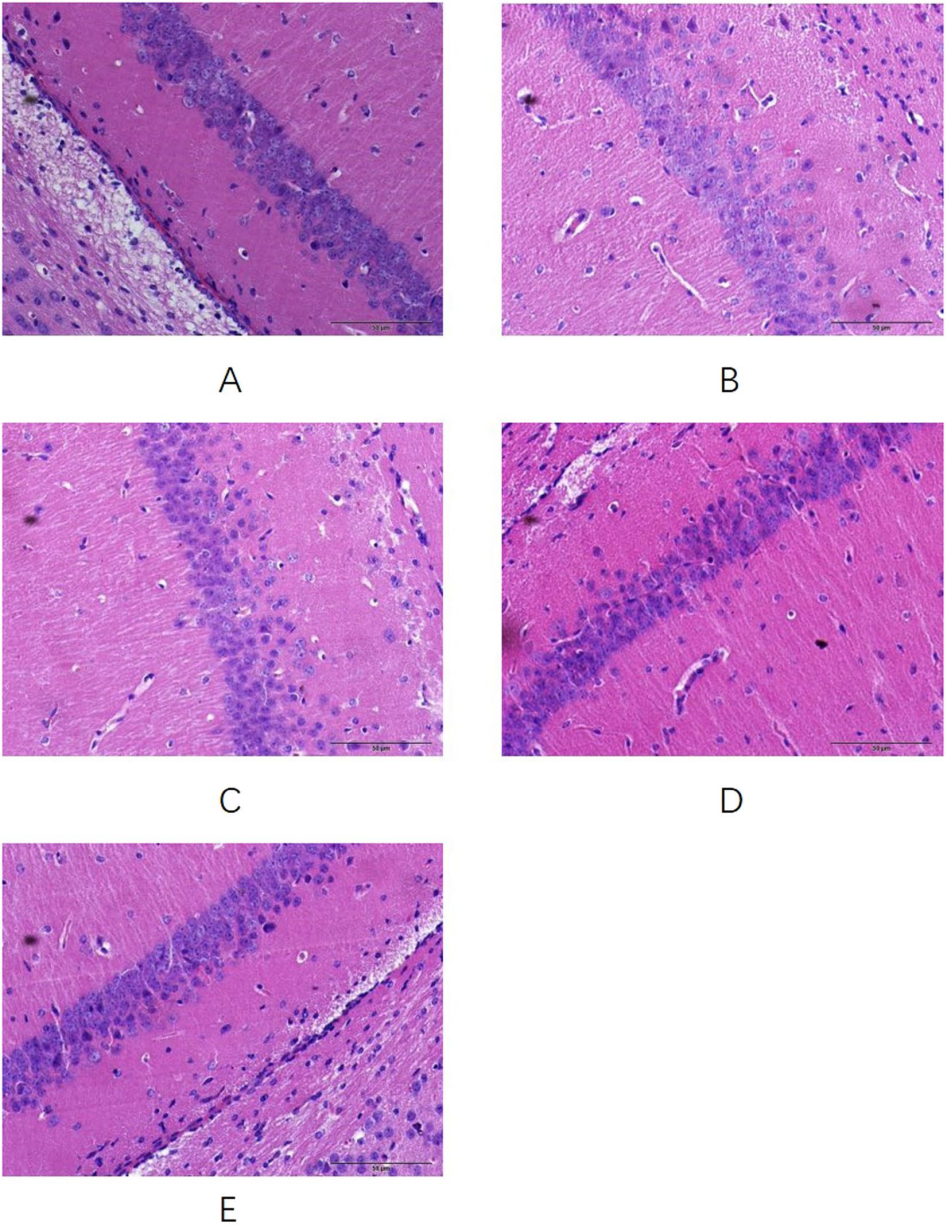
Effects of SCE administration on the histopathological changes in the hippocampus of depressed mice. The scale bar shows 50 μm. Control (**A**), CUMS (**B**), SCE 300 mg/kg (**C**), 600 mg/kg (**D**) and 1200 mg/kg (**E**).

H.E. staining was performed to detect the neuronal integrity and orderliness. In the hippocampus, the neurons appeared intact and ordered, shrinkage, degeneration and necrosis of the nuclei were not observed in the control mice (Fig. 9A), but shrinkage of nuclei, swollen and eccentrically dispersed neuronal bodies were found in the CUMS mice (Fig. 9B). However, administration of SCE could inhibit the above mentioned histopathological damages significantly (Fig. 9C,D,E).

## Discussion

**Figure 10:**
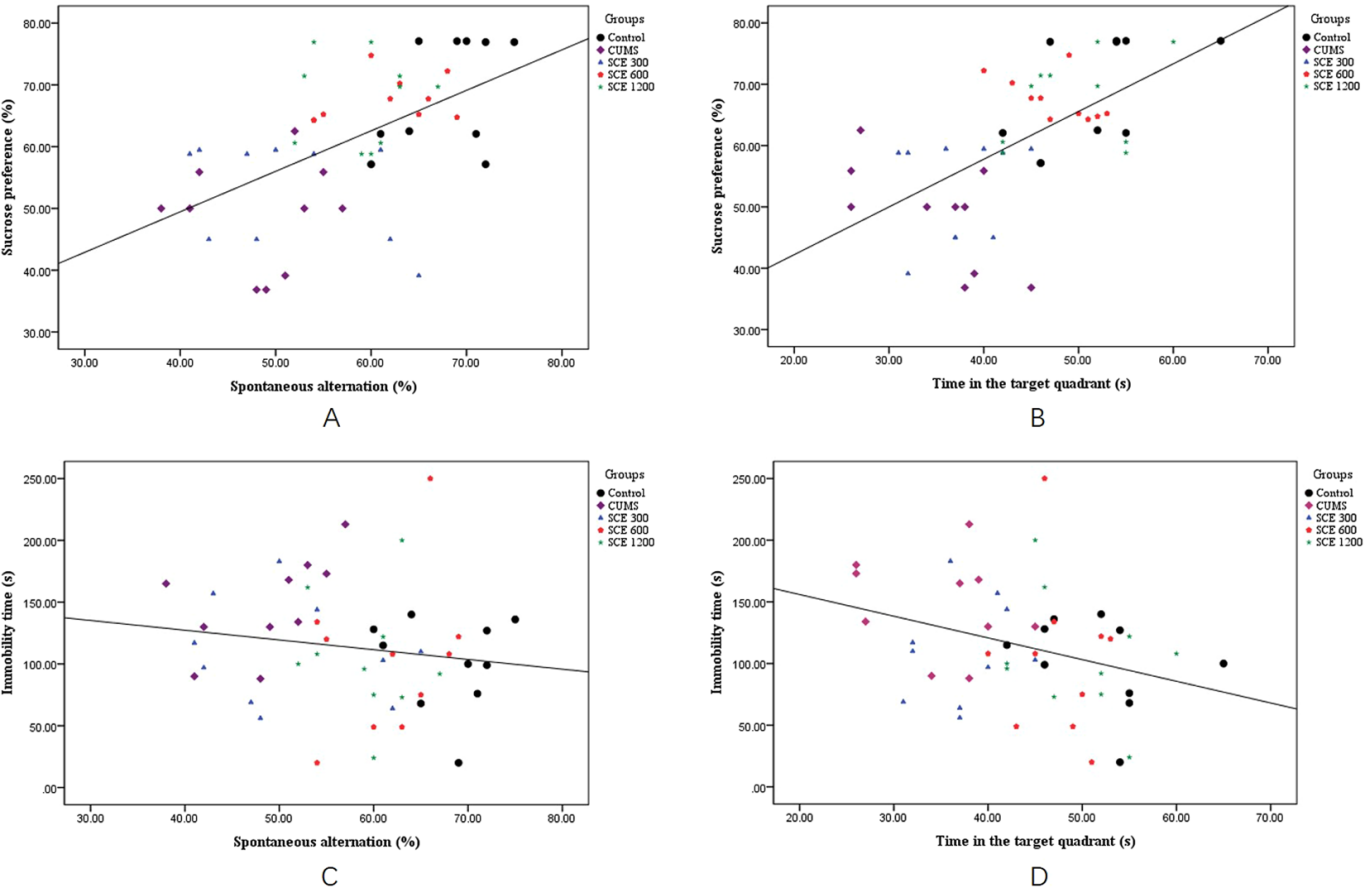
Pearson’s correlation between the sucrose preference vs. spontaneous alternation (**A**), the sucrose preference vs. time in the target quadrant (**B**), immobility time vs. spontaneous alternation (**C**) and immobility time vs. time in the target quadrant (**D**).

Numerous neuropsychological studies have examined the role of cognition in psychopathology, revealing that most psychiatric disorders are associated with some degree of cognitive impairment^32^. We used CUMS procedure to mimic the symptom of depression confirmed by SPT and FST in mice in our behavioral studies, which is consistent with previous reports^33^. Further, CUMS also impaired the cognitive function of mice according to the performances of Y maze and MWM test, which are two classical methods to assess the ability of learning and memory. These results are well coincident with previous studies^34, 35^. However, with the treatment of SCE, the anhedonia-like behavior in sucrose preference test and immobility time in forced swim test which both induced by CUMS were significant increased, respectively. On the other aspect, the spontaneous alternation in Y maze, time in the target quadrant and escape latency in training of SCE mice were remarkable improved compared to the CUMS mice. And No significant differences of the total travel distance in the locomotor activity test and number of arm entries in Y maze which could exclude the false positive results caused by the mobility of mice during all the behavioral tests. Moreover, we found a significant correlation between the sucrose preference vs. spontaneous alternation, the sucrose preference vs. time in the target quadrant, immobility time vs. spontaneous alternation and immobility time vs. time in the target quadrant when linear regression was determined (Fig. 10). Integrated with the results of H.E. staining, we found that SCE indeed has the ability to improve the depressive symptoms and the associated cognitive deficits effectively.

The interrelationship between depression and cognitive impairment is complex and still not well understood. In the brain, BDNF has been implicated in development, neural regeneration, synaptic transmission, synaptic plasticity and neurogenesis^36^. Alternation in BDNF levels have been implicated in psychiatric disorders, including depression and substance abuse, as well as neurodegenerative disorders, such as Alzheimer’s, Parkinson’s and Huntington’s diseases^37^. We found that in the hippocampus of CUMS-induced mice, BDNF levels were significantly decreased, and the results of Y-maze and MWM test were shown that the cognition impairment along with the depression symptoms. After treatment with SCE, the BDNF levels were upregulated, and the depression symptoms and cognition decline were alleviated at the same time according to the behavioral results. Besides the results of immunohistochemistry analysis and Immunofluorescence assay, the above all illustrated that BDNF has played a vital role both in depression and the learning obstacle triggered by it, which in line with the previous reports^38^.

**Figure 11:**
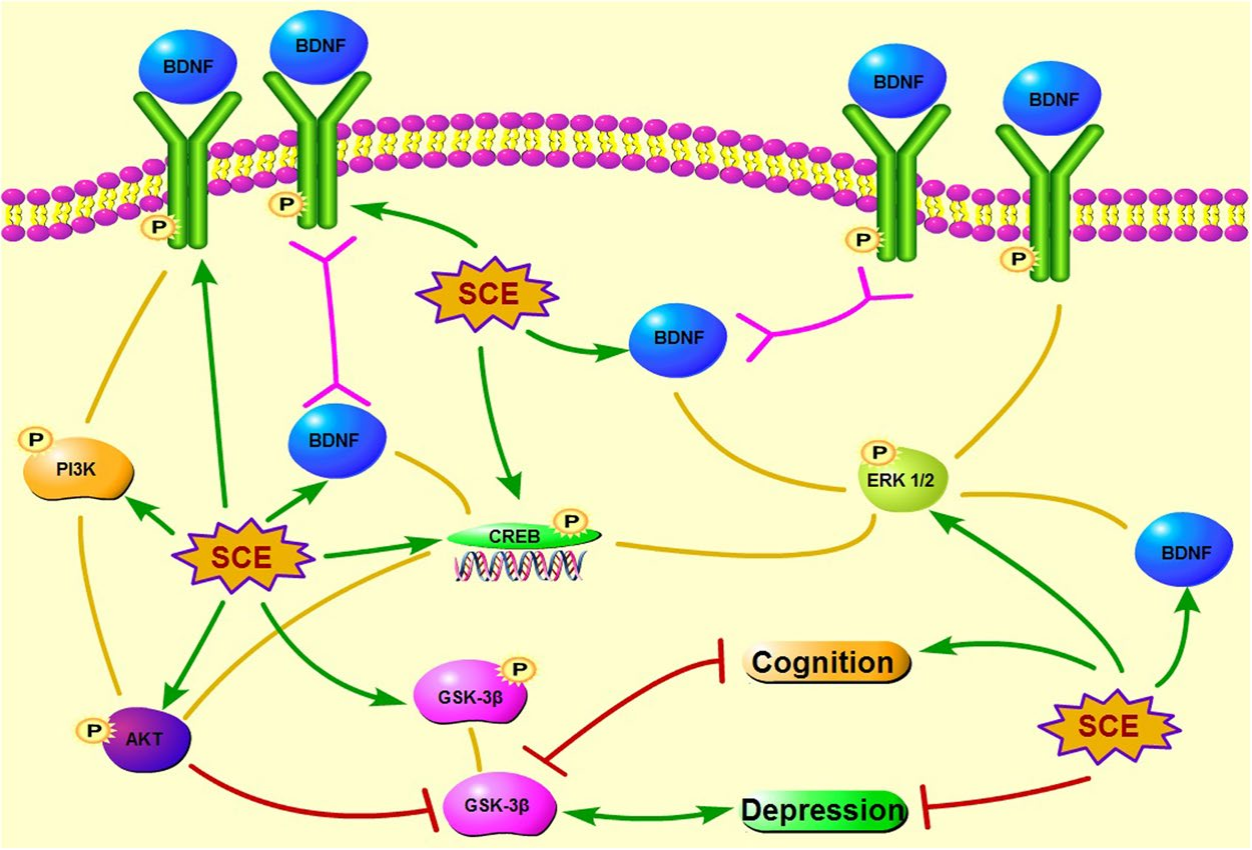
Proposed SCE antidepressant-like mechanisms for the interaction among BDNF, TrkB, CREB, ERK, PI3K, AKT and GSK-3β. SCE regulates TrkB/CREB/ERK pathway to increase the BDNF to alleviate the depression and cognitive decline. On the other aspect, BDNF upregulates PI3K/AKT/ GSK-3β pathway to ameliorate the depressive symptoms and cognition disability induced by CUMS.

Tropomyosin-related kinase B (TrkB), a protein tyrosine kinase receptor and a member of the larger family of Trk receptors, which was expressed at high levels in the brain, was identified as the primary signal transduction receptor for BDNF. Multiple lines of evidence link BDNF-TrkB signaling to the pathophysiology of MDD, as well as the therapeutic mechanisms of antidepressants^39, 40^. Furthermore, extensive experimental evidence supports that BDNF mitigates depressive symptoms mainly by binding to TrkB, leading to autophosphorylation of TrkB tyrosine residues, and activation of downstream signaling molecules, including the extracellular signal-regulated kinase 1/2 (ERK1/2) known to phosphorylate cAMP response element binding protein (CREB)^41^. It has been reported that the full-length TrkB autophosphorylation regulate ERK on activation by BDNF, which may increase cAMP and activate CREB-regulated gene transcription, and this mechanism further promotes transcription of BDNF^42^. In agreement with previous studies, we observed that CUMS-induced depressive symptoms and consequent BDNF/TrkB/CREB/ERK1/2 signaling down-regulation reversed by SCE treatment, which indicated that SCE could increase BDNF expression by affecting TrkB/CREB/ERK1/2 pathway to exert antidepressant-like effect. Additionally, interaction of BDNF with TrkB triggers receptor dimerization, transphosphorylation of intracellular tyrosine residues, and subsequent activation of the three major signaling pathways involving MAPK/CREB/ERK, phosphatidylinositol 3 kinase (PI3K)/AKT, and phospholipase C-γ^43^. PI3K/AKT and ERK signaling pathways are the major TrkB-mediated survival pathways that promote neuronal survival and protect against apoptosis. BDNF/TrkB signaling can promote further BDNF production through CREB via activation of PI3K/AKT or ERK signaling, which is emerging as a positive-feedback loop^44^. TrkB-induced PI3K activity leads to AKT activation, and once activated, AKT leads to inactivation of GSK-3β at Ser-9. More recent findings showed that disruption of GSK-3 phosphorylation by AKT decreases anxiety and reduces proneness to depression in mice^45^ and intracellular signal transduction system as AKT/GSK-3β pathway have been found to be altered in the brain of depressive patients^46^. This provides compelling evidence that the PI3K/AKT/GSK-3β signaling pathway is an important contributor to depression. Here we demonstrated that the SCE treatment was able to activate PI3K/AKT/GSK-3β signaling pathway, as well to promote increased phosphorylated levels of related proteins, to improve the depressive syndromes in CUMS-induced mice. Taken all above together, these findings lead us to suggest that SCE acting as an antidepressant increases BDNF levels by promoting TrkB/CREB/ERK pathway and activating PI3K/AKT/GSK-3β pathway simultaneously (Fig. 11).

Based on the former research, depression can be a psychological reaction to cognitive decline, and thus may also appear as an early symptom in dementing individuals^47^. However, recent data suggest that depression, and in particularly late-life depression, can also be a risk factor for Alzheimer’s disease^48^. In our present study, we confirmed that SCE could treat depression and show a definite improvement of cognition. The further and in-depth study on the interactions between depression and dementia, and the separate, simultaneous effects of SCE on these two kinds of diseases would be performed in the future study.

In summary, our study shows that the antidepressant-like effect and cognition improvement ability of SCE depends on BDNF levels raise by increasing TrkB/CREB/ERK pathway and PI3K/AKT/GSK-3β pathway in the meantime. Our findings suggest that SCE might be a promising therapeutic agent of depression, and further research is worth to be invested.

## Methods

### Preparations of extract of SCE

The fruits of SCE were purchased from the TCM shop of Tongrentang (Shenyang, China) and identified by Professor Ying Jia (Department of Pharmacognosy, Shenyang Pharmaceutical University) according to the guidelines of the Chinese Pharmacopoeia (2015). Then, the fruits of SCE were exhaustively extracted with 95% ethanol at reflux for 2 h 3 times. After concentration in a vacuum, the residue was suspended in 0.5% sodium carboxymethycellulose (CMC-Na) at a certain concentration of 300 mg/kg, 600 mg/kg or 1200 mg/kg.

### UPLC–Q-TOF/MS analysis of SCE

**Figure 12:**
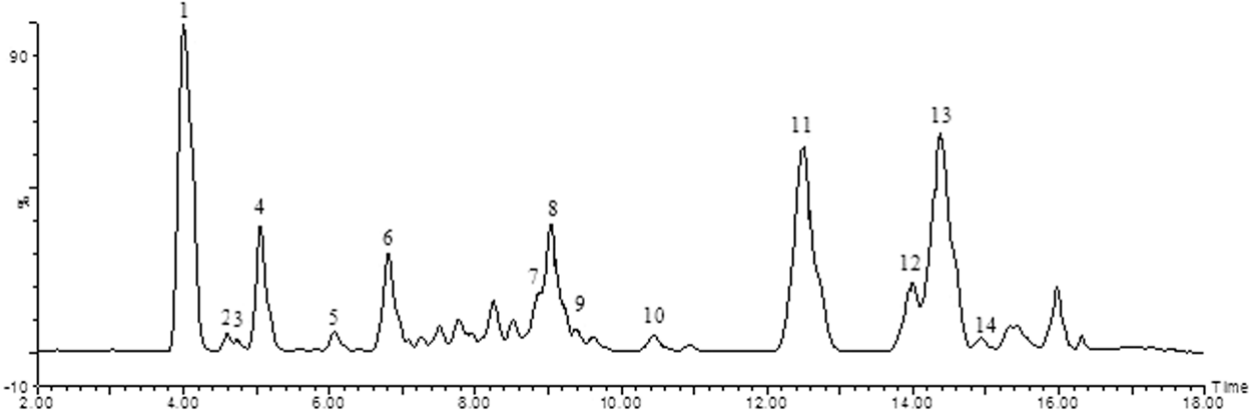
UPLC chromatogram of SCE (1) schisandrin; (2) gomisin D; (3) gomisin J; (4) schisandrol B; (5) tigloylgomisin H; (6) angeloylgomisin H; (7) schisantherin A; (8) schisantherin B; (9) Schisanhenol; (10) gomisin E; (11) deoxyschizandrin; (12) gomisin N; (13) Schisandrin B; (14) Schisandrin C.

The chemical composition of SCE was analyzed by using a Waters-UPLC-Q-TOF/MS with an ultraviolet/visible detector (UV/Vis) coupled to an ion trap mass spectrometer with an ESI interface. The separation was achieved on an HSS T3 Column (100 mm × 2.1 mm, 1.8 μm). The chromatogram was recorded at 216 nm. Mass analyses were performed using an ESI interface in the positive ion mode. Data were performed with Masslynx V4.1 software. As shown in Fig. 12 and Table 3, fourteen lignans were tentatively identified by the full scan on the positive ion mode of MS/MS analysis. Eight main compounds (1, 4, 7, 8, 11, 12, 13 and 14) of those lignans were identified with the retention time and UV spectra of the reference substance.

**Table 3:** Tentative identification of the compounds from SCE.

Peak	*T*_*R*_ (min)	MS (*m/z*)	Major fragments (m/z)	Tentative identification
1	4.00	432.2148	433.2227[M + H]^+^, 415.2125[M + H - H_2_O]^+^	Schisandrin
2	4.60	530.2152	531.2224[M + H]^+^, 548.2492[M + NH4]^+^	Gomisin D
3	4.74	388.1886	389.1964[M + H]^+^	Gomisin J
4	5.04	416.1835	399.1804[M + H - H_2_O]^+^	Schisandrol B
5	6.08	500.2410	501.2477[M + H]^+^, 483.2390[M + H - H_2_O]^+^	Tigloylgomisin H
6	6.81	500.2410	501.2484[M + H]^+^, 483.2390[M + H - H_2_O]^+^	Angeloylgomisin H
7	9.01	536.2046	537.2094[M + H]^+^	Schisantherin A
8	9.04	514.2203	515.2275[M + H]^+^	Schisantherin B
9	9.37	402.2042	403.2116[M + H]^+^	Schisanhenol
10	10.44	514.2203	515.2275[M + H]^+^	Gomisin E
11	12.51	416.2199	417.2284[M + H]^+^, 439.2108[M + Na]^+^	Deoxyschizandrin
12	14.00	400.1886	401.1962[M + H]^+^	Gomisin N
13	14.38	400.1886	401.1962[M + H]^+^	Schisandrin B
14	14.94	384.1573	385.1651[M + H]^+^, 407.1471[M + Na]^+^	Schisandrin C

### Chemicals and Reagents

Fluoxetine hydrochloride as a positive control drug was obtained from Melone Pharmaceutical Co. (Dalian, China). All other chemicals and reagents were of analytical grade.

### Animals

Adult male Kunming mice (weighing 20 ± 2 g) were purchased from the Experimental Animal Center of Shenyang Pharmaceutical University (Shenyang, China). All of them were maintained under standard laboratory conditions of constant temperature (23 ± 1 °C), relative humidity (50 ± 10%) and a 12 h light/dark cycle (light from 7:00 a.m. to 7:00 p.m.) with food and water available ad libitum and were allowed to habituate to the novel environment for 1 week prior to use in experiments. The experiment was carried out in compliance with the National Institutes of Health and institutional guidelines for the humane care of animals and was approved by the Animal Care Committee of Shenyang Pharmaceutical University (Protocol No.: SYPU-IACUC-2016C-0921-205). Every effort was made to minimize the number of animals used and any pain and discomfort experienced by the subjects.

### CUMS procedure

CUMS was performed as previously described^49^. CUMS consisted of exposure to a variety of unpredictable stressors (randomly), including (1) 24 h food deprivation, (2) 24 h water deprivation, (3) 1 h exposure to an empty bottle, (4) exposure to an empty cage (without sawdust bedding), (5) grouped housing, (6) 24 h soiled cage (200 ml water in 100 g sawdust bedding), (7) level shaking for 30 min, (8) 5 min cold swimming (at 5 °C), (9) nip tail for 1 min. All stresses were applied individually and continuously, day and night. The control animals were housed in a separate room and had no contact with the stressed groups. To prevent habituation and to ensure the unpredictability of the stressors, all stresses were randomly scheduled, repeated throughout the 3-week experiment. The control group mice were left undisturbed except for necessary procedures such as routine cage cleaning.

### Drug administration and experimental groups

**Figure 13:**
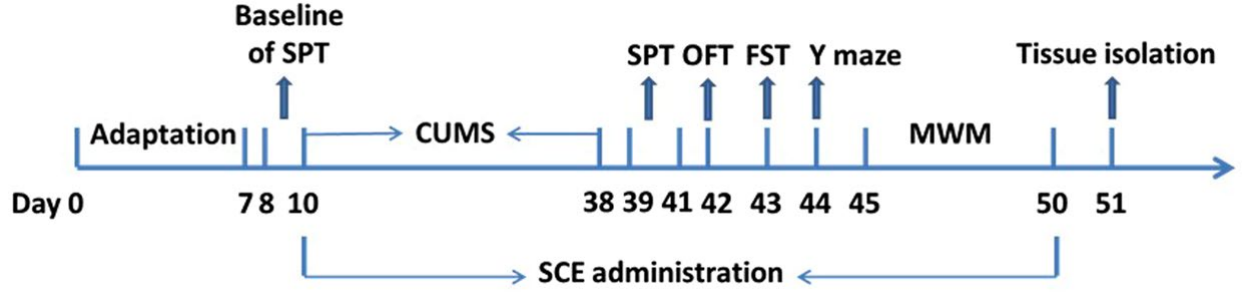
Schematic representation of time-line for SCE treatment in CUMS induced expetimental animals.

For testing of the behavioral and BDNF regulations, the mice were randomly divided into six groups (10 animals for each group): Control-Vehicle group, CUMS-Vehicle group, CUMS-fluoxetine (10 mg/kg, i.g.), CUMS-SCE group (300 mg/kg, i.g.), CUMS-SCE group (600 mg/kg, i.g.) and CUMS-SCE group (1200 mg/kg, i.g.). For the Control-Vehicle group, the animals were injected with same amount of 0.5% CMC-Na. All these agents were administered in a volume of 10 ml/kg. Before CUMS, animals were allowed to habituate to all the behavioral tests in order to establish an individual baseline. The sucrose preference tests were conducted twice before and after the CUMS procedure, respectively. Before the tissue isolation, the mice were allowed to take the test of all the behavioral tests. Behavioral tests were performed during the light phase of light–dark cycle. The whole experimental procedure is shown in Fig. 13.

### Behavioral analyses

#### Sucrose preference test (SPT)

Sucrose preference test was carried out 1 day before CUMS and the end of CUMS. The test was performed as described previously^50^. In brief, 72 h before the test, the mouse was individually placed in a cage with two bottles of sucrose solution to adapt a sucrose solution (1%, w/v) for 24 h; then one bottle of sucrose solution was replaced with water (24 h); after the adaptation, laboratory mice were deprived of water and food (24 h). Sucrose preference test was conducted at 9:30 a.m. Each mouse was housed in individual cage and free to access two bottles containing 200 ml of sucrose solution (1% w/v) and 200 ml of water, respectively. After 6 h, the consumed weights of sucrose solution and water were recorded. The sucrose preference value was obtained from the following formula: sucrose preference (%) = sucrose intake (ml)/[sucrose intake (ml) + water intake (ml)] × 100%.

### Inner open-field behavior test (OFT)

In order to rule out the possibility that the alteration in the immobility time in the FST was due to interference of the locomotor activity, spontaneous locomotor activity of each mouse was observed in an open filed experimental video analysis system (ZS-ZFT, Huaibei Zhenghua Bio-Apparatus Co. Ltd, China). The apparatus was placed in a darkened and sound attenuated testing room. The total path of Spontaneous locomotive was evaluated over a 5 min period^51, 52^.

### Forced swim test (FST)

The FST was conducted and the total immobility was estimated in accordance with the methods described previously^53^ with slight changes. In brief, each mouse was forced to swim in a cylindrical glass container (diameter 20 cm × height 50 cm) with 30 cm of water (22 ± 1 °C) for 6 min, and the duration of immobility during the last 4 min was detected. The mice were considered as immobile when they maintained floating in the water without escaping from the container, making only the movement necessary to keep their heads above the water. The parameter was recorded by trained observers who were blind to the experimental groups.

### Y-maze test

Y-maze test as a measure of immediate spatial working memory which is a form of short-term memory^54^. The Y-maze is consisted of three arms at equal angles (30 cm length × 5 cm width × 12 cm high). Mice were placed at the end of one arm and allowed to move freely through the maze for 6 min. An arm entry was counted when the hind paws of the mouse were completely within the arm. The series of arm entries were recorded visually and the percentage alternation was calculated. A spontaneous alternation was defined as successive entries into the three arms, i,e., ABC, CAB, or BCA but not CBC. The percentage alternation was calculated as the ratio of actual to possible alternations (defined as the total number of arm entries minus two) multiplied by 100 as shown by the following equation: Alternation% = [Number of alternations/(Total arm entries − 2)] × 100. The number of arm entries was also used as an indicator of locomotor activity.

### Morris water maze test (MWM)

A spatial learning and memory test was performed by the method of Morris with minor difference^55^. The Morris water maze consisted of a large circular tank (120 cm in diameter, 40 cm in height) which was divided geographically into four quadrants (I, II, III and IV). A black platform (9 cm in diameter, 30 cm in height) was submerged 1 cm below the water surface and fixed at the midpoint of the IV quadrant. The water was colored with non-toxic black ink and was maintained at 23 ± 2 °C. The tank was placed in a dimly lit, soundproof test room with various visual cues for navigation. The Morris water maze test was composed of training trials and probe test. Before test, each animal was screened in MWM according to the speed and swimming state, and a white plastic platform was used to evaluate the visible ability of mice. In the training trials, the mice were placed in the water facing the pool wall from two different starting points every day and allowed to swim freely to seek the hidden platform for a maximum 90 s. If a mouse failed to find the platform within 90 s, it was guided by an experimenter to the platform to study 30 s. The training trials lasted five consecutive days. The average escape latency of each mouse per day was recorded. The probe test was performed without platform after the 5-day training finished. The percentage of time spent of swimming in the target quadrant and the times crossed where the platform was originally located were measured for each mouse^56^.

### Biochemical analysis

#### Tissue sample collection

After the completion of the behavioral test, the mice were euthanized. Whole brains were rapidly removed from the mice and stored according to the specific experimental procedure. Brain regions of the hippocampus were dissected on a cold plate and immediately frozen in liquid nitrogen. The tissue samples were stored at −80 °C until assay.

### ELISA kits assay

BDNF, pTrkB, TrkB, Pcreb, CREB, pERK and ERK concentrations of hippocampus determination were performed using the ELISA method, according to the mouse ELISA kits manufacturer’s instructions (Shanghai Enzyme-linked Biotechnology Co., Ltd, China), respectively. Optical density was obtained at 450 nm using a microplate reader (Varioskan flash, Thermoscientific, USA) within 15 min of stop solution addition.

### Western blot

Hippocampal tissues were homogenized in RIPA (150 mM sodium chloride, 50 mM Tris (pH 8.0), 0.5% sodium deoxycholate, 0.1% SDS, 1% Triton X-100) and PMSF (Dalian Melonepharma, China) and kept on ice for 30 min. The tissue homogenate was centrifuged at 10 000 g for 20 min at 4  °C. The supernatant was obtained and used as the total hippocampal and cerebral cortex protein extract measured by BCA assay kit (Dalian Melonepharma, China) to determine protein concentration and stored at −80 °C until use.

Samples were diluted with an equal volume of loading buffer (Beyotime Biotech Co., China), and boiled at 95 °C for 5 min. Approximately 50 μg of protein was loaded in each well and separated in 10% or 12% SDS-PAGE gels. The proteins were transferred onto nitrocellulose membranes. The membranes were saturated and blocked with 5% fat-free powdered milk at 37 °C for 1.5 h and incubated overnight at 4 °C in one of the following primary antibodies, which were diluted in 5% fat-free powdered milk in TBS: PI3 Kinase p85 (19H8) (1:1000, CST, USA), Akt (pan) (C67E7) (1:1000, CST, USA), GSK-3β (D5C5Z) (1:1000, CST, USA), GADPH Rabbit mAb (1:1000, CST, USA), β-actin Rabbit mAb (1:1000, CST, USA). Phospho- GSK-3β (Ser 9) (1:1000, CST, USA), Phospho-Akt (Ser473) (1:2000, CST, USA). Anti-PI3K p85 (phosphor Y607) (1:1000, Abcam, USA) Blots were washed three times for 30 min in TBST at room temperature and then incubated for 1.5 h in one of the following HRP-conjugated antibodies, which were diluted in 5% fat-free powdered milk in TBS: Anti-rabbit IgG (1:2000, CST, USA) for detection of target proteins, GADPH and β-actin. After three times washes for 30 min in TBST, immunolabeled protein bands were detected using the ECL Western blot detection kit (Dalian Melonepharma, China). Graphs of blots were obtained in the linear range of detection and were quantified for the level of specific induction by scanning laser densitometry.

### Immunohistochemistry assay

Mice were sacrificed by overdose of sodium pentobarbitone for immunohistochemical analysis, and then intracardially perfused with PBS, followed by chilled 4% PFA in PBS. The brain was sliced into 15 mm sections on a cryostat blocked in PBS containing 1% goat serum and 0.1% Triton × 100, and incubated at 4 °C overnight with anti-BDNF (mouse IgG, 1:500, abcam, USA). After washing, a HRP conjugated goat anti-rabbit IgG complex (1:1000, absin, China) was applied for 1 h. Color development was performed with a diaminobenzidine peroxidase substrate kit (TL-125-QHD, Thermo Fisher Scientific, USA). Sections were conunterstained with hematoxylin.

### Immunofluorescence assay

Tissue biopsies were deparaffinized and permeabilized with PBS/0.1% Triton × 100. Antigen retrieval was performed by boiling the slides in 0.01 M trisodium citrate buffer, pH 6, for 10 min. Sections were then preincubated with 10% normal goat serum containing 0.2% Triton × 100 overnight at 4 °C to block nonspecific binding. Slides were then incubated over night at 4 °C with anti-BDNF (mouse IgG, 1:100, abcam, USA) in 2% serum, sections were washed three times in PBS to remove unbound primary antibody, then incubated with secondary antibody for 1 h at 37 °C. After that, sections were washed three more times in PBST (PBS, with 1% Tween 20) and covered with DAPI/Fluorescence quenching agent (Beyotime, China). Slides were viewed using a Leica fluorescence microscope coupled with a computer assisted video camera (Axioscope A1, Germany).

### Histology assay

For histopathology, hematoxylin-eosin and Congo red were used as described previously^57^. After the behavioral tests animals were decapitated and their brains were removed quickly, post fixed in 37% formaldehyde solution for 48 h, and then all the brain was cut in the coronal plane and stained with hematoxylin and eosin. Hippocampus neurons were examined in light microscopy.

### Statistical analysis

Results are expressed as mean ± SEM. The significances between different groups were assessed using one-way ANOVA, followed by Tukey HSD post-hoc test when significant main effects were indicated. Two-way ANOVA was used to analyze data from the Morris water maze training trials. In all calculations, p < 0.05 was considered to be statistically significant. Statistical analysis was performed with SPSS software 19.0.
